# Supplementary material for: Mitochondrial Peptide Humanin Facilitates Chemoresistance in Glioblastoma Cells
Source: Cancers (Basel). 2023 Aug 11;15(16):4061. doi: 10.3390/cancers15164061 (PMC10452904; doi:10.3390/cancers15164061)

SUPPLEMENTARY FIGURE

Supplementary Figure S1: HN mRNA expression in human GBM cells treated with chemotherapy

Human GBM cells (U251) were incubated with or without cisplatin (5 µM) for 48 h. HN mRNA expression levels were quantified using qPCR. The gel shows bands corresponding to specific HN amplification in response to chemotherapy.

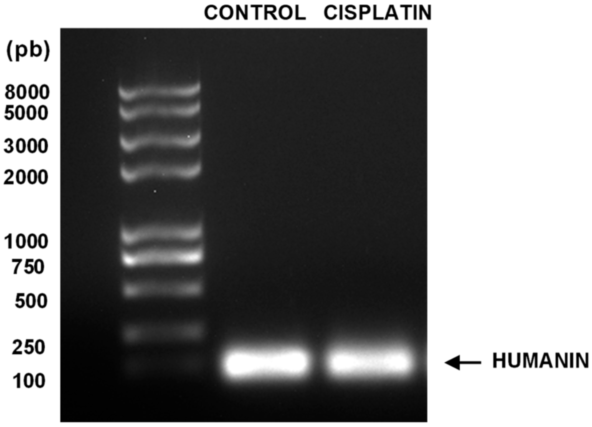

Supplementary Figure S2. Zimography of active MMPs in HNGF6A-treated GBM cellconditioned media

SDS-PAGE gelatin zymography of conditioned media from human GBM U251-MG cells incubated in the presence of HNGF6A (1.25 µM) for 48 h (n = 3 replicates/condition). The table indicates the densitometric value of each band as assessed with the ImageJ software (Version: 1.53k). The zymographic activity was expressed as a percentage in relation to a standard internal sample that is saturated at a density of 50%. A representative gel is shown.

SAMPLE	%MMP-2	%MMP-9
Internal control	100.00	100.00
CONTROL 1	126.83	5.99
CONTROL 2	117.90	6.75
CONTROL 3	145.46	8.38
HNGF6A 1	164.51	8.21
HNGF6A 2	167.72	7.86
HNGF6A 3	136.64	8.94

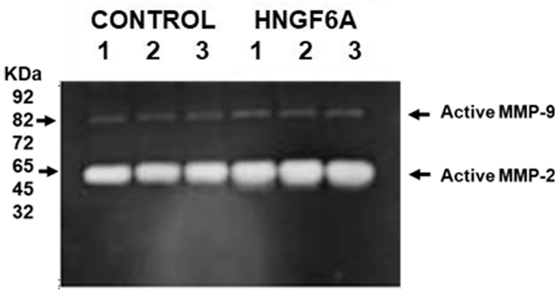

Supplementary Figure S3: Chemotherapy up-regulates HN expression in murine GBM cells

Murine Gl26 GBM cells were incubated with 5 µM cisplatin for 48 h. HN expression was assessed by immunofluorescence. Images show cells immunostained with HN antibody (red), and DAPI-stained nuclei (blue).

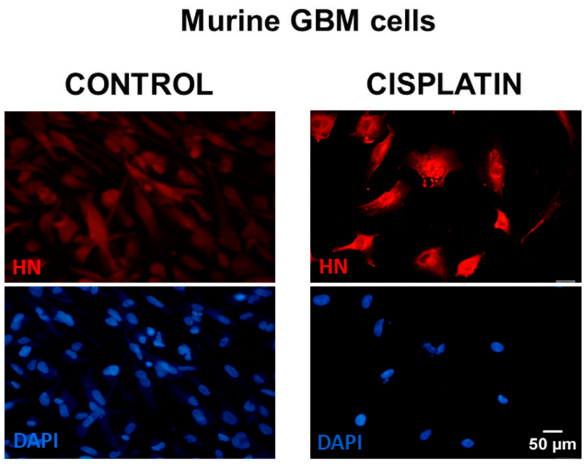

Supplement: Supplementary file 1 [file cancers-15-04061-s001.zip › cancers-2516190-supplementary.pdf]
